# Effect of different ulnar osteotomies on loading of the distal radioulnar joint: a finite element analysis

**DOI:** 10.1186/s12891-024-07562-3

**Published:** 2024-06-08

**Authors:** Jiyang Tan, Fei Zhang, Qianyuan Liu, Xiaodong Fang, Hong Jiang, Jun Qian, Jingyi Mi, Gang Zhao

**Affiliations:** 1 Medical College, Soochow University, Suzhou, China; 2 Department of Sports Medicine, Wuxi 9th People’s Hospital Affiliated to Soochow University, Liangxi Road No. 999, Wuxi, Jiangsu, China; 3 Department of Hand Surgery, Wuxi 9th People’s Hospital Affiliated to Soochow University, Liangxi Road No. 999, Wuxi, Jiangsu, China

**Keywords:** Ulnar impingement syndrome, Ulna osteotomy, Distal radioulnar joint, Distal oblique bundle, Stress

## Abstract

**Background:**

Ulnar impingement syndrome is a prevalent source of ulnar carpal pain; however, there is ongoing debate regarding the specific location of shortening, the method of osteotomy, the extent of shortening, and the resulting biomechanical alterations.

**Method:**

To investigate the biomechanical changes in the distal radioulnar joint (DRUJ) resulting from different osteotomy methods, a cadaveric specimen was dissected, and the presence of a stable DRUJ structure was confirmed. Subsequently, three-dimensional data of the specimen were obtained using a CT scan, and finite element analysis was conducted after additional processing.

**Results:**

The DRUJ stress did not change significantly at the metaphyseal osteotomy of 2–3 mm but increased significantly when the osteotomy length reached 5 mm. When the osteotomy was performed at the diaphysis, the DRUJ stress increased with the osteotomy length, and the increase was greater than that of metaphyseal osteotomy. Stress on the DRUJ significantly increases when the position is changed to pronation dorsi-extension. Similarly, the increase in stress in diaphyseal osteotomy was greater than that in metaphyseal osteotomy. When the model was subjected to a longitudinal load of 100 N, neither osteotomy showed a significant change in DRUJ stress at the neutral position. However, the 100 N load significantly increased stress on the DRUJ when the position was changed to pronation dorsi-extension, and the diaphyseal osteotomy significantly increased stress on the DRUJ.

**Conclusions:**

For patients with distal oblique bundle, metaphyseal osteotomy result in a lower increase in intra-articular pressure in the DRUJ compared to diaphyseal osteotomy. However, it is crucial to note that regardless of the specific type of osteotomy employed, it is advisable to avoid a shortening length exceeding 5 mm.

## Introduction

Ulnar impaction syndrome is a prevalent condition that often leads to ulnar pain [1]. The two commonly employed treatments for this syndrome are ulnar shortening osteotomy (USO) and arthroscopic wafer surgery [2]. The USO technique involves two types of osteotomy: diaphyseal and metaphyseal ulnar shortening osteotomies [3]. Surgeons typically select surgical procedures based on their personal preferences and experience, without any objective criteria for selection. Previous studies have mainly focused on the surgical technique and the extent of osteotomy. However, it is equally important to understand the stability and biomechanical changes of the distal radioulnar joint (DRUJ) after osteotomy. When osteotomy is performed in the proximal or distal oblique bundle (DOB) [4], the pulling effect of the DOB can lead to difficulties in shortening or even insufficient shortening [5]. Sayuri Arimitsu’s [6] research demonstrated that proximal osteotomy greatly enhanced the stability of the DRUJ. However, it remains unclear whether proximal osteotomy can also increase surface stress on the radioulnar joint and potentially lead to premature joint degeneration in patients with already stable joints. To investigate this hypothesis, the present study conducted finite element analysis on cadaver specimens.

## Method

In this study, we used 1 fresh-frozen cadaver upper extremity that was amputated at the midportion of the humerus. Our objective was to determine the stress condition of bones after osteotomy using the finite element method for stress analysis. The analysis process involved acquiring image data, establishing a grid model, reconstructing a geometric model, and conducting finite element analysis. CT scanning was used to obtain wrist image information, Mimics Medical 21.0 was utilized for grid acquisition, Geomagic Design X was employed for geometric model reconstruction, and Ansys Workbench 19.0 was used for finite element (FEM) analysis.

### Anatomical analysis of cadaver specimens

**Fig. 1:**
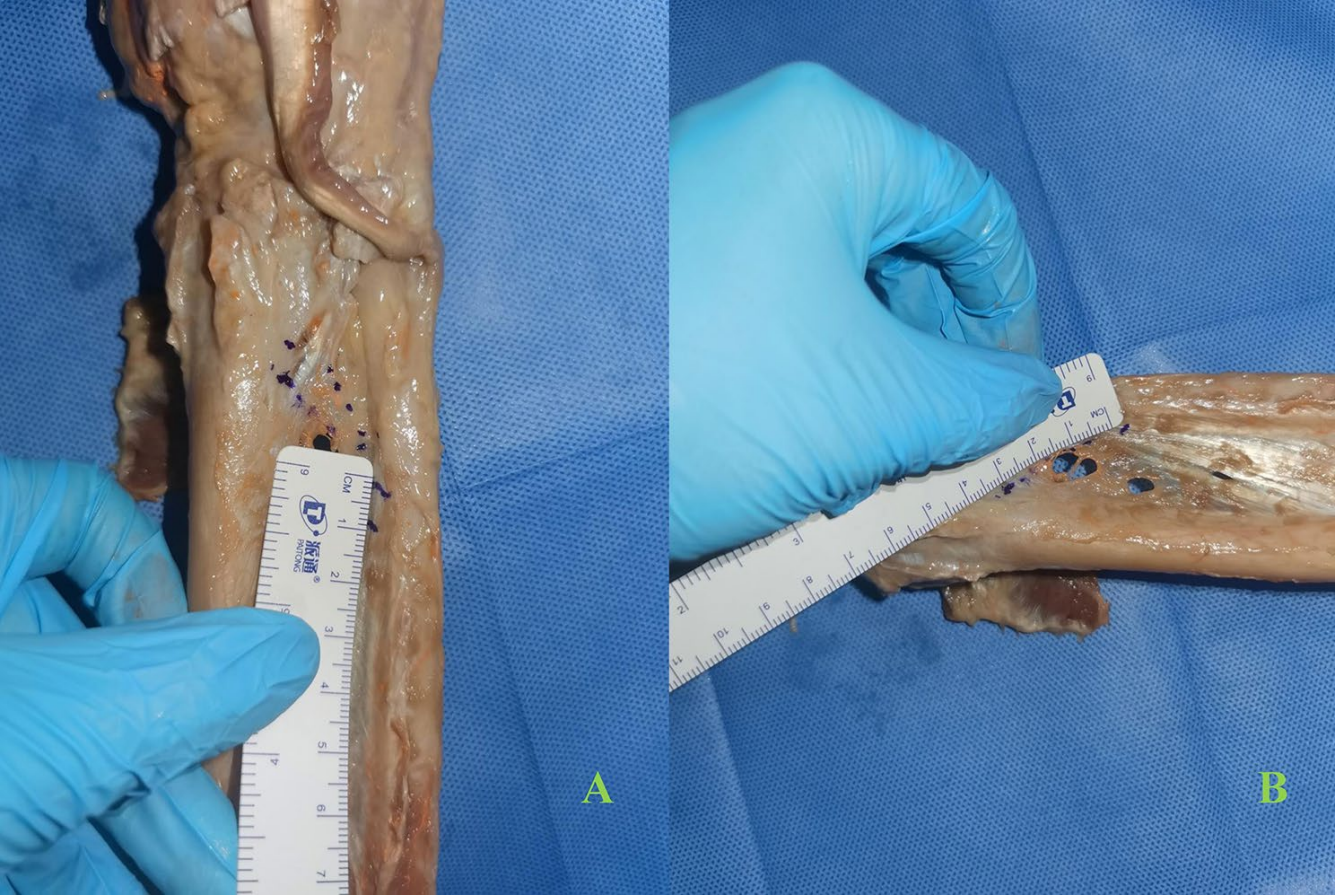
DOB of the specimen exists, with a width of 8 mm (**A**) and a length of 3.5 mm (**B**), which is also the basis for DOB reconstruction in the FEM model

The selected cadaver specimen was dissected, revealing the presence of DOB (Fig. 1). Additionally, we examined the integrity of other stable structures, such as the triangular fibrocartilage complex (TFCC), extensor carpi ulnaris (ECU)sheath, and ulnarcollateral ligament (UCL), which were found to be intact in the specimen. The starting and ending points, as well as the lengths, of all ligaments required for wrist joint finite element model reconstruction are measured and recorded. These include the long/short radiolunate, radiocapitate, radioscaphoid, ulnocapitate, ulnolunate, ulnotriquetral, palmer radioulnar, medial anterior, medial posterior, lateral radial, lateral ulnar, and distal oblique bundle.

### Establishment of the FEM model

**Fig. 2:**
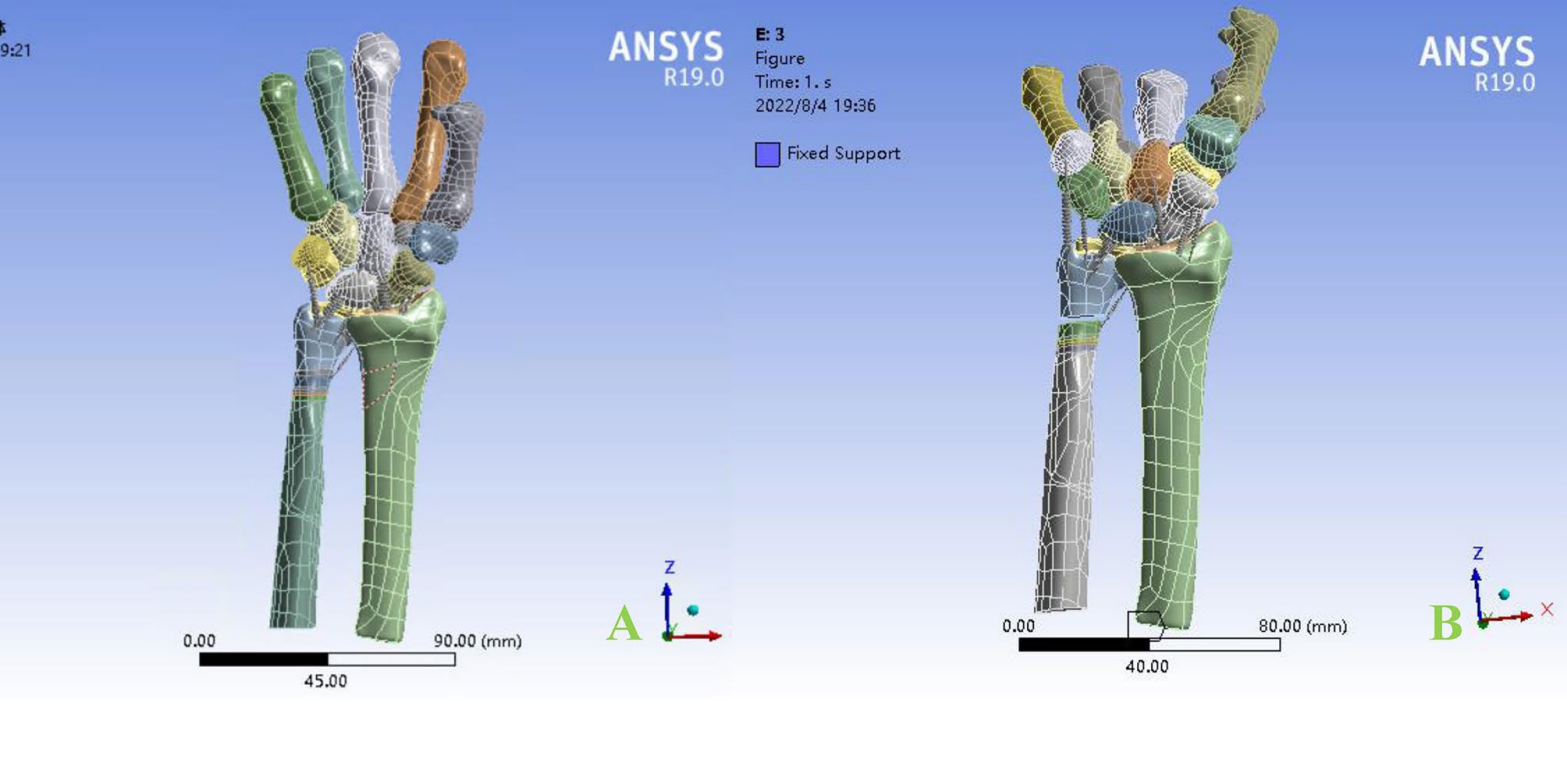
A geometric model for analysis: **A**: neutral position; **B**: dorsi-extension position

The selected specimens were positioned in a 64-slice CT scanner (GE, USA) in a neutral position. The CT scanning parameters were set as follows: 120 ~ 140 kV, 525 mA, and a layer thickness of 0.625 mm. The scan was performed from the metacarpophalangeal to the forearm discontinuity. Subsequently, the specimens were adjusted to the pronation dorsi-extension position for another CT scan. The DICOM format data of both positions were imported into Mimics21.0 software (Materialise, Belgium) for reconstruction of the DRUJ model. The reconstructed model was then imported into Geomagic Design X software for surface treatment and optimization of model characterization. The generated model was further imported into Ansys Workbench 19.0 to create the carpal bone geometric model for FEM analysis. Additionally, the required carpal cartilage model was created in Spaceclaim, and the carpal ligament was constructed using spring units based on the wrist’s structure, as shown in Fig. 2.

**Fig. 3:**
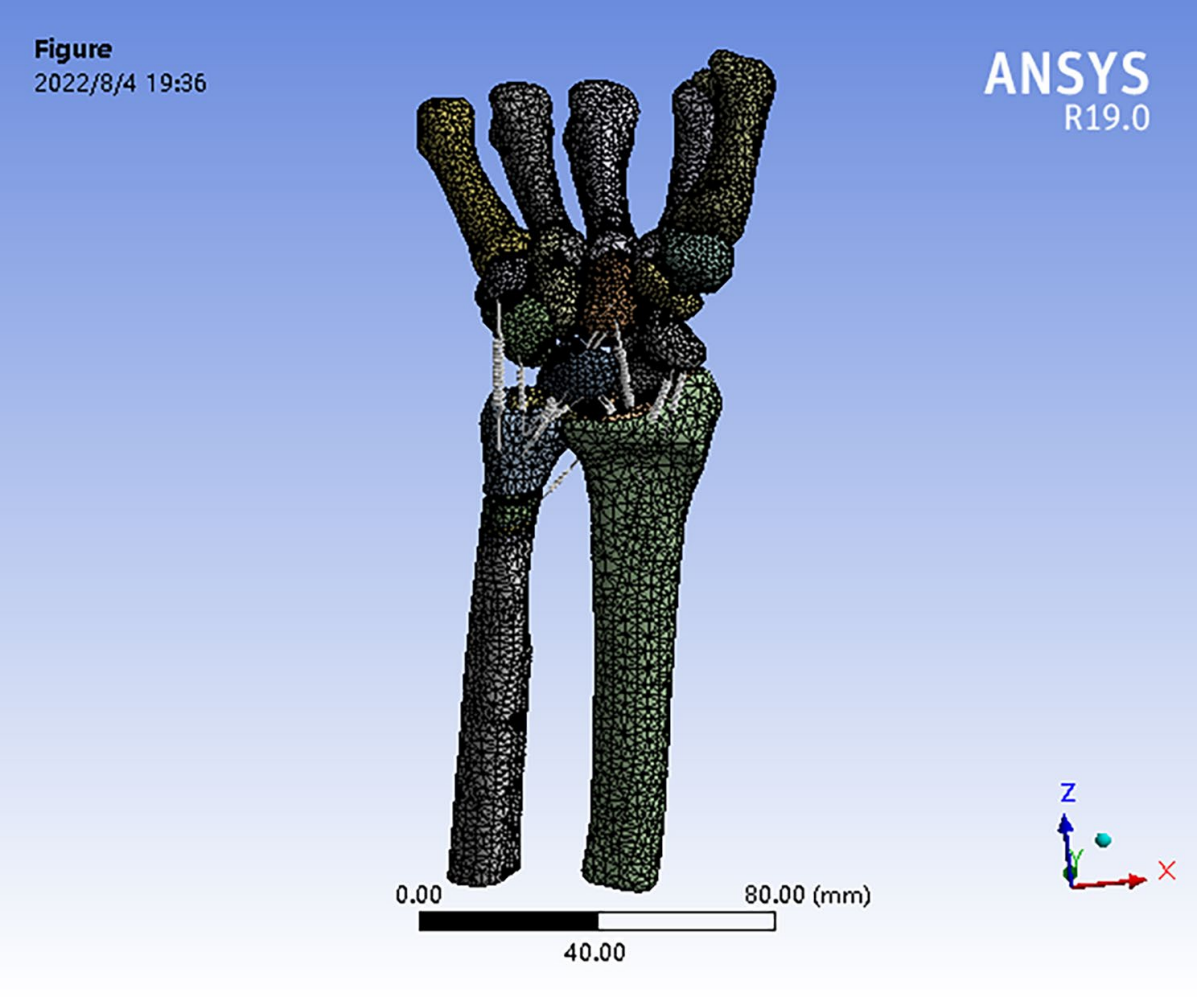
Second-order tetrahedral mesh is selected to map the geometric model with a mesh size of 2 mm

### Map the geometric model

To map the geometric model, a second-order tetrahedral mesh with a mesh size of 2 mm was chosen, as depicted in Fig. 3.

### Materials, loads and boundary conditions

The material properties of cortical bone are as follows: the density is 1600 kg/m-3, Young’s modulus is 12,000 MPa, and Poisson’s ratio is 0.3. Cartilage has a Young’s modulus of 10 MPa and a Poisson’s ratio of 0.45. The ligament parameters, load, and boundary conditions are summarized in Table 1, which were taken from previously published works [7–9]. A simulated osteotomy was performed on the finite element model of the wrist joint in both the neutral and pronation dorsi-extension positions. Osteotomies of 2 mm, 3 mm, and 5 mm were performed at the ulnar metaphysis and ulnar diaphysis. The DRUJ was recorded under nonweight-bearing and 100 N conditions to determine the maximum contact stress and distribution position of cartilage under axial stress. The results are presented as a cartilage equivalent stress nephogram, and the data were recorded.

**Table 1:** Ligament properties used in the model

Ligament	Abbreviation	Stiffness(*N*/mm)
Long/Short radiolunate	LRLL/SRLL	40.0
Radiocapitate	RCL	50.0
Radioscaphoid	RSL	50.0
Ulnocapitate	UCL	50.0
Ulnolunate	ULL	40.0
Ulnotriquetral	ULL	40.0
Palmer radioulnar	PRUL	11.0
Medial anterior	MAL	72.3
Medial posterior	MPL	52.2
Later radial	LRL	15.5
Later ulnar	LUL	57.0
Distal oblique bundle	DOB	18.9

### Result

**Fig. 4:**
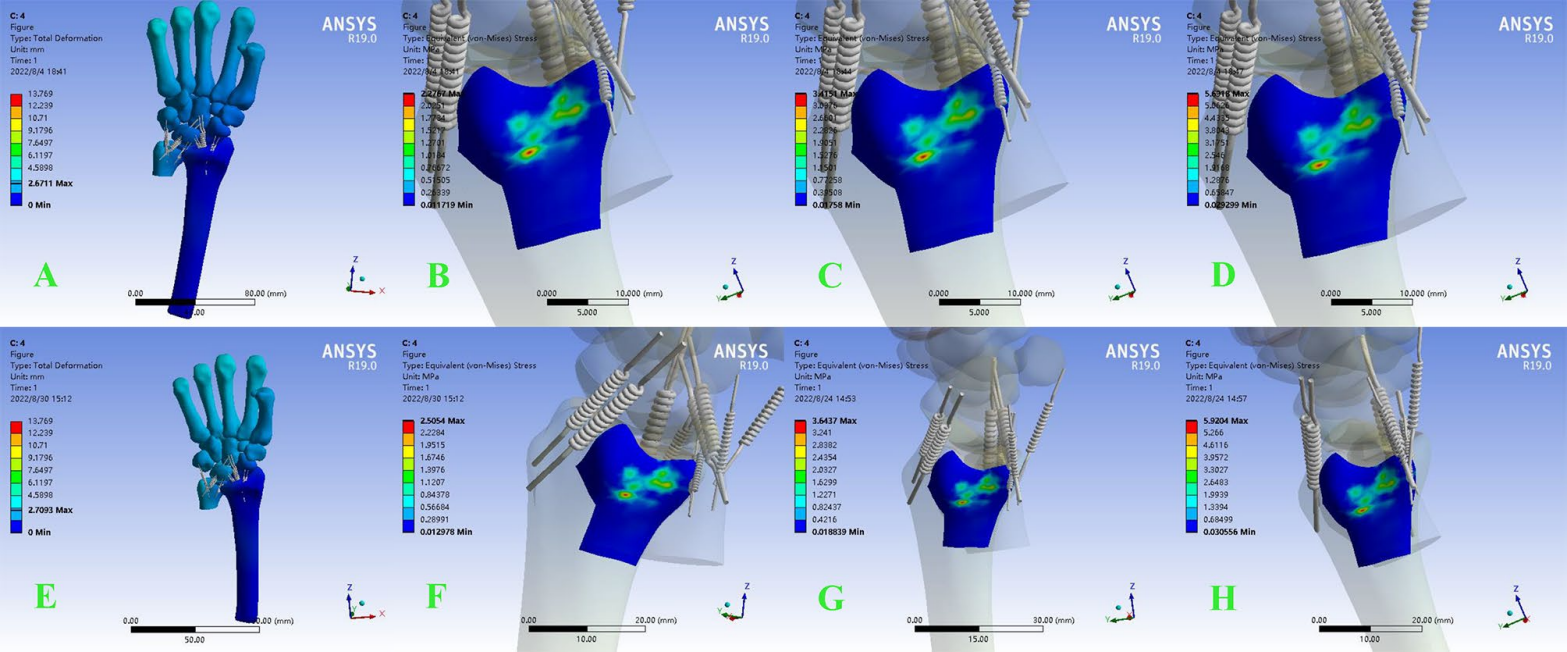
In metaphyseal osteotomy cartilage equivalent stress cloud diagram of DRUJ in neutral position. **A ~ D** is in non-load-bearing state when shortened by 2,3,5 mm , **E ~ H** is under axial stress when shortened by 2,3,5 mm. The red area in the figure indicates the maximum contact stress, and the dark blue area indicates the minimum contact stress. The same as the figure below

When the osteotomy was located at the metaphysis, there was no significant change in stress on the articular surface of the DRUJ at 2–3 mm neutral and pronated dorsi-extension. However, when the osteotomy length reached 5 mm, the force on the DRUJ surface increased significantly, and the increasing trend was more obvious in the pronation dorsi-extension position. When a force of 100 N was applied to the metacarpal, the neutral DRUJ had little change in force compared with the unapplied force. However, stress on the DRUJ increased at 100 N in pronation and was more pronounced at 5 MM osteotomy length (Fig. 4).

**Fig. 5:**
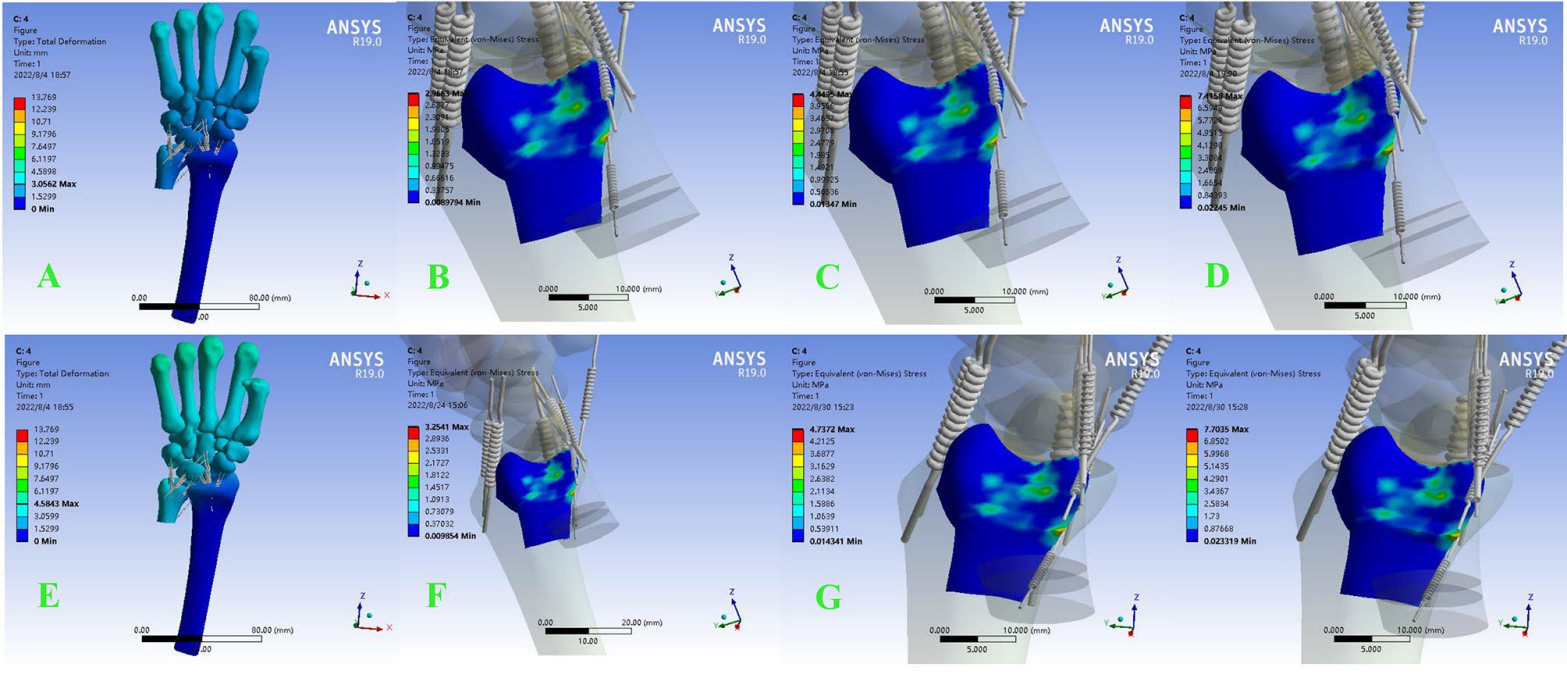
In diaphyseal osteotomy, cartilage equivalent stress cloud diagram of DRUJ in neutral position. **A ~ D** are in non-load-bearing state when shortened by 2,3,5 mm. **E ~ H** are under axial stress when shortened by 2,3,5 mm

When the osteotomy was located at the diaphysis, the stress on the DRUJ increased as the length of the osteotomy increased. The increasing trend of DRUJ stress at a neutral position was not influenced by longitudinal pressure. However, when the position changes to pronation dorsi-extension, the stress on the DRUJ surface increases as the longitudinal pressure increases ( Fig. 5).

**Fig. 6:**
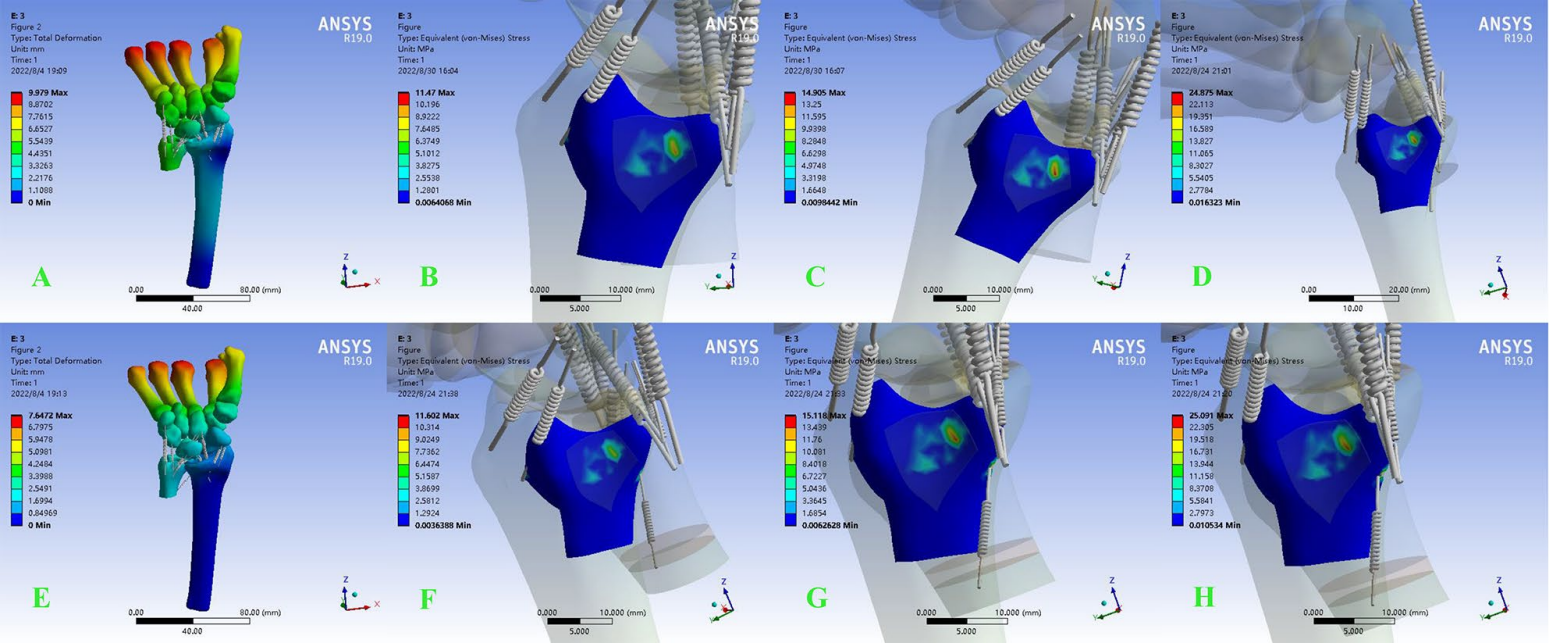
The DRUJ stress in pronation dorsi-extension, e (**A-D**: metaphyseal osteotomy 2,3,5 mm. **E-H**: Diaphyseal osteotomy 2, 3, 5 mm)

The tension of the DOB ligament was further increased in pronation dorsi-extension due to the change in the position of the ulna relative to the radius. Thus, a 100 N load applied in the pronated dorsi-extension resulted in a dramatic increase in DRUJ stress, and a significant increase was observed in both osteotomy methods (Fig. 6).

The comparison between the two groups revealed that DRUJ stress increased as the length of the osteotomy increased, regardless of the specific length of the osteotomy when it was performed at the diaphyseal region. Notably, metaphyseal osteotomy resulted in a significant increase in DRUJ stress only at a length of 5 MM. On the other hand, the diaphyseal osteotomy caused greater stress on the DRUJ in the supination dorsi-extension position compared to the metaphyseal osteotomy. Additionally, when a longitudinal pressure of 100 N was applied, the rate of increase in stress remained consistent. Detailed data are shown in Table 2.

**Table 2:** Stress(Mpa) of DRUJ with different osteotomy lengths, type and position under two loads

Load(N)	Osteotomy length(mm)	neutral		dorsi-extension	
		metaphyseal	diaphyseal	metaphyseal	diaphyseal
0	2	2.276	2.966	7.478	7.979
	3	3.415	4.449	11.715	11.968
	5	5.692	7.416	18.944	19.946
100	2	2.709	2.982	11.47	11.602
	3	3.861	4.387	14.905	15.118
	5	6.455	7.383	24.875	25.091

## Discussion

Ulnar impingement syndrome causes pain on the ulnar side of the wrist due to increased pressure on the ulnocarpal joint. Ulnar shortening osteotomy is an effective surgical procedure to relieve this pressure and has definite clinical benefits [10]. Currently, it is the most commonly performed surgery for ulnar impingement syndrome (UIS). However, after the shortening procedure, the tension on the ligaments related to the ulnocarpal joint increases, which improves the stability of the distal radioulnar joint (DRUJ) [11]. On the other hand, this increased tension also leads to higher contact stress on the DRUJ, potentially causing degeneration of the osteocartilage and even arthritis [12]. According to literature reports, the incidence of DRUJ osteoarthritis after ulnar diaphysis shortening ranges from 17–34% [11, 13]. Ulnar shortening surgery can be classified into diaphyseal and metaphyseal osteotomy, with the main biomechanical difference being the presence or absence of the interosseous membrane, particularly the mechanical involvement of the distal oblique band (DOB). The distal oblique bundle (DOB) is a solid structure of the distal interosseous membrane (DIOM). It extends from the distal one-sixth of the ulna shaft, around the level of the proximal margin of the pronator quadratus, to the proximal border of the dorsal rim of the ulnar notch of the radius [14]. The DOB is partially connected to both the volar and dorsal portions of the radioulnar ligament of the triangular fibrocartilage complex (TFCC). It is widely acknowledged that the DOB plays a crucial role in maintaining the stability of the distal radioulnar joint. Studies have indicated that the distal oblique bundle experiences the least change in length during forearm rotation, suggesting that it acts as an isometric stabilizer for the distal radioulnar joint. The metaphyseal osteotomy plane is located far from the DOB. On the one hand, it is not restricted by the interosseous membrane, making shortening easier. On the other hand, unlike diaphyseal osteotomy, which increases DOB tension, metaphyseal osteotomy does not affect DOB tension. This can, to some extent, reduce the contact stress on the DRUJ and potentially decrease the incidence of DRUJ. Additionally, metaphyseal osteotomy is easy to perform and benefits from a rich blood supply at the fracture site, which is crucial for bone healing. Our team’s published clinical studies have demonstrated that metaphyseal osteotomy leads to quicker pain relief and faster bone healing [15]. A retrospective meta-analysis of 6 clinical studies highlighted a higher secondary operation rate for diaphyseal osteotomy compared to metaphyseal osteotomy, with diaphyseal osteotomy also showing inferior pain relief [16]. Additionally, a study involving 69 patients found that metaphyseal osteotomy provided significantly better pain relief outcomes than diaphyseal osteotomy [17]. As a result, metaphyseal osteotomy has gained increasing popularity in clinical practice in recent years.

In this study, a finite element model was developed to analyze the change in DRUJ contact stress during simulated metaphyseal and diaphyseal osteotomy of the wrist joint in neutral and pronation dorsal extension positions. The findings indicated that DRUJ contact stress increased regardless of the presence of load, with a more significant increase observed in cases of diaphyseal osteotomy. These results are consistent with the findings of Graybe et al. [18], who also reported an increase in DRUJ contact stress following ulnar osteotomy. The analysis suggests that the increase in DRUJ stress after metaphyseal osteotomy may be attributed to the increase in soft tissue tension, such as the ligaments surrounding the ulnar carpal joint [19], which are not influenced by the distal interosseous membrane and therefore show a relatively smaller increase. According to a study by Lapner et al., the reduction in the DRUJ contact area can increase its contact stress. When the ulna is shortened by 5 mm, the DRUJ contact stress increases by 200% [20]. In this study, when the osteotomy length was greater than 5 mm, the DRUJ contact stress increased significantly (greater than 300%). As a result of this long-term effect, it may lead to degenerative changes in DRUJ osteocartilage and accelerate the occurrence of DRUJ osteoarthritis. In a finite element analysis conducted by Farzaneh Gholamian et al. [21] it was observed that DRUJ stress rises proportionally to the applied torque during forearm rotation. Another study by Desney Greybe et al. [22] indicated that an increase in the anteversion of the radius can result in up to a 20% increase in DRUJ stress.Additionally, taking into account previous studies [23–26], this paper selected the pronated dorsiflexion position. This choice was primarily based on the understanding that ulna positive variation increases when the wrist is in the pronated dorsiflexion position, which in turn increases the load on the ulnocarpal joint compared to the neutral position and decreases the DRUJ contact area. Consequently, it can increase the contact stress of the DRUJ to a certain extent. Furthermore, dorsiflexion of the wrist joint can further increase the contact stress of the DRUJ by tensing the ulnocarpal ligament, particularly after axial stress [27], as confirmed by the results.

Further research on the optimal osteotomy volume is still needed. Nishiwaki et al. [11] confirmed that shortening the ulna by 3–6 mm can increase TFCC tension and stabilize the DRUJ. Biomechanical research by Palmer and Werner [28] has shown that shortening the ulna by 2.5 mm can reduce the load on the distal ulnar bone by 14%, providing significant relief to the ulnar wrist joint. However, excessive osteotomy can lead to problems. First, due to ligament limitations, it can be difficult for the ulnar bone to slide to the proximal end and achieve reduction. Forced pulling may damage the stable structures of the DRUJ, such as the TFCC and DOB, resulting in DRUJ instability. Second, ulna shortening can increase the contact stress on the DRUJ. Biomechanical studies have confirmed that when ulna shortening exceeds 3 mm, the DRUJ contact stress significantly increases [18]. The study found that chondrocyte apoptosis began when the compressive stress exceeded 4.5 MPa [29]. The results suggest that when the metaphysis of the diaphysis of the neutral ulna is shortened by 4 mm, the contact stress of the DRUJ is close to this value. However, when pronation and dorsal extension are shortened by 2 mm, the contact stress exceeds this value. Therefore, it is believed that ulnar osteotomy larger than 4 mm may increase the risk of DRUJ cartilage injury. This paper suggests that effective decompression of the ulnar carpal joint should be considered without excessively increasing DRUJ contact stress. Complete correction of ulnar positive variation is not necessary. Additionally, in pronation and dorsal extension positions, the contact stress of the DRUJ dorsal region is significantly increased, especially under axial loading. Therefore, it is recommended to reduce weight-bearing activities involving pronation and dorsal extension after clinical ulnar shortening to protect the articular cartilage.

Although this study conducted a quantitative study on the change regularity of DRUJ contact stress after osteotomy, the results have certain clinical significance. However, there are some limitations to consider. First, this study did not take into account the influence of different DRUJ shapes on the contact stress after osteotomy. Second, the computational model is derived from a cadaver specimen that has been dissected to verify the integrity of specific structures, and the absence of muscle tone and any alterations from the dissection process may impact the correlation between anatomical structures and the application of results in clinical settings. Finally, The model used in this study has a DOB ligament, which may not be present in all individuals. Therefore, the conclusions drawn from this study are only relevant to individuals with a DOB ligament. Further research will be conducted to investigate the clinical characteristics of individuals with DOB ligament deficiency.

## Conclusion

Based on the results of finite element analysis, we believe that for patients with DOB ligament, metaphyseal osteotomy leads to a lower increase in DRUJ intra-articular pressure compared with diaphyseal osteotomy, which can potentially reducing the risk of secondary osteoarthritis. However, it is important to note that regardless of the type of osteotomy, it is advisable to avoid shortening the length by more than 5 mm. Additionally, patients should be cautious about exerting excessive force in the pronation dorsi-extension position after surgery to prevent injury to the articular surface cartilage of the DRUJ.

## Data Availability

The datasets used or analyzed during the current study are available from the corresponding author upon reasonable request.
